# Dietary habits among snus users: a population-based cross-sectional study

**DOI:** 10.29219/fnr.v67.9537

**Published:** 2023-08-21

**Authors:** Fanny Berglund, Johanna Törmä, Maria Wennberg, Patrik Wennberg, Viktor Oskarsson

**Affiliations:** 1 Department of Health and Medical Services, Region Norrbotten, Luleå, Sweden; 2 Department of Public Health and Caring Sciences, Uppsala University, Uppsala, Sweden; 3 Department of Public Health and Clinical Medicine, Umeå University, Umeå, Sweden

**Keywords:** tobacco, smokeless tobacco, snus, diet, food, beverages

## Abstract

**Background:**

The dietary habits among snus users are largely unknown and have not been accounted for in observational studies on the health effects of snus use.

**Aim:**

To examine whether snus users eat unhealthier than never tobacco users.

**Methods:**

A total of 3,397 male participants, examined between 1994 and 2014 in the Northern Sweden Monitoring of Trends and Determinants in Cardiovascular Disease (MONICA) study, were included. Snus use and dietary habits were self-reported using questionnaires, from which intakes of different food groups, macronutrients, and a healthy diet score (HDS) were calculated (the latter as a proxy for overall diet quality). The association between snus use and dietary habits was examined by quantile regression models.

**Results:**

In the multivariable-adjusted model, current snus users had a lower HDS (median difference: −0.86 [95% confidence interval: −1.32, −0.40]) than never tobacco users. Snus users also consumed fewer weekly servings of fruits and berries (median difference: −1.03 [−1.65, −0.40]), and their estimated percentage of energy intake consisted of less carbohydrates (median difference: −1.43 [−2.12, −0.74]) and of more total fat (median difference: 0.99 [0.30, 1.67]), saturated fat (median difference: 0.67 [0.29, 1.05]), monounsaturated fat (median difference: 0.44 [0.20, 0.68]), trans fat (median difference: 0.03 [0.01, 0.06]), and alcohol (median difference: 0.21 [0.02, 0.40]).

**Conclusion:**

We observed that snus users had an unhealthier diet than never tobacco users. Future studies on the association between snus use and health outcomes should, therefore, consider diet as a potential confounder.

## Popular scientific summary

- Snus is a common tobacco product in Scandinavian countries. However, the dietary habits among snus users are largely unknown and have not been accounted for when examining the health effects of snus use.
- In the current study, it was observed that snus users had an unhealthier diet than never tobacco users with respect to certain food groups and macronutrients as well as to overall diet quality.
- Future studies on the association between snus use and health outcomes should, therefore, consider diet as a potential confounder.

Snus is a smokeless tobacco product that is commonly used in Scandinavian countries, especially among men. In 2021, the male-specific prevalence of snus use was 20% in Sweden (1), 19% in Norway (2), and 7% in Finland (3). In general, snus use is associated with less negative health effects than cigarette smoking (as reviewed by Hajat et al. [4]), which can be exemplified by the absence of a clear association between snus use and incidence of cardiovascular disease (CVD) (5–15). However, several studies have observed that snus use might lead to an increased mortality in CVD (12–14, 16). (Nota bene: the epidemiological litterature on the association of snus use with CVD incidence and mortality is summarized in **Online Resource 1**).

It should be noted that none of the studies on the association of snus use with CVD incidence and mortality has accounted for the dietary habits among snus users. A poor diet is an established risk factor for CVDs (17, 18) and, as such, a possible confounder for the association between snus use and CVD outcomes. According to the European Heart Network, dietary risk factors have the largest contribution of behavioral risk factors to CVD mortality and CVD disability-adjusted life years, with an estimation that up to 49.7% of the male-specific CVD mortality in Sweden is attributable to diet (19). Interestingly enough, the dietary habits of snus users have seldom been studied. One Norwegian study found that snus users were less likely to have regular eating habits than their non-using peers (20), and a Swedish study found that snus users were less likely to have regular breakfast habits and to be daily consumers of fruit and berries compared with non-tobacco users (21). In contrast, it is well-established that cigarette smokers have unhealthier diets than non-tobacco users with respect to both macronutrients (exemplified by, among others, higher intakes of total and saturated fat and lower intakes of polyunsaturated fat and fiber) (22) and overall diet quality (exemplified by a higher dietary energy density [energy per gram of food]) (23). Although speculative at this point, it is possible that the dietary habits of snus users could be similar to those of cigarette smokers.

Using data from the Northern Sweden Monitoring of Trends and Determinants in Cardiovascular Disease (MONICA) study, the aim of this study was to examine the dietary habits among snus users, particularly in comparison with never users of tobacco products.

## Subjects and methods

### Study population

The Northern Sweden MONICA study consists of seven population-based surveys that were conducted in Västerbotten and Norrbotten between 1986 and 2014. Each survey used a random sampling design, stratified for sex and age (25 to 64 years in 1986 and 1990 and 25 to 74 years in 1994 to 2014). The participation rate decreased over time, from 81% in 1986 to 63% in 2014. Participants answered a questionnaire on sociodemographic and lifestyle factors (available in Swedish at www.umu.se/forskning/projekt/monica-studien/monica-undersokningen-i-norra-sverige2/), answered a food-frequency questionnaire (FFQ), and underwent a clinical examination (24).

The current study was restricted to male participants, due to the low number of female snus users in the Northern Sweden MONICA cohort (around 5%), and to participants examined between 1994 and 2014, due to the use of a different FFQ in the surveys in 1986 and 1990.

The Northern Sweden MONICA study has been covered by multiple ethical approvals from the Regional Ethical Committee at Umeå University (Sweden) from its initiation up until 2014 (of which 2013/97-31 is of most relevance for the current study). A written informed consent was obtained from all participants.

### Assessment of tobacco use and other variables

In the questionnaire on sociodemographic and lifestyle factors, the participants answered a number of questions related to their current and previous use of tobacco (i.e. snus, cigarettes, pipe, cigars, and cigarillos). To minimize confounding by current or previous use of multiple tobacco habits, the following categories of tobacco use were applied: ‘never tobacco’ (no history of tobacco use), ‘past tobacco’ (past history of tobacco use), ‘current snus’ (current snus use), ‘current smoking’ (current or occasional smoking of cigarettes, pipe, cigars, or cigarillos; of which cigarettes accounted for 85.2% of the total use), and ‘current snus and smoking’ (current snus use and current or occasional tobacco smoking).

The participants also answered questions on educational level, civil status, and history of diabetes. Height and weight were measured during the clinical examination, and body mass index (BMI) was calculated as weight (kg) divided by height squared (m^2^).

### Assessment of diet

Dietary intake was assessed using a semi-quantitative FFQ, which contained 84 questions on a 9-point scale (from ‘never’ to ‘>4 times/day’). Portion sizes were specified for potatoes/rice/pasta, meat/fish, and vegetables using photo illustrations of four different portion sizes. Reported food intake frequencies were converted to grams per day based on data from a validation study (25), and energy and nutrient intakes were estimated by multiplying food intake frequencies with portion sizes as well as with data on nutrient contents from the Swedish Food Agency (26). Macronutrients (e.g. carbohydrates, protein, and fat) were converted to percentages of daily energy intake by multiplying the daily intake of each macronutrient (in grams) with its caloric value (e.g. 9 kilocalories [kcal] per gram of fat) and dividing that number by the daily energy intake (in kcal).

A healthy diet score (HDS) was calculated as a proxy for the overall diet quality (27, 28). The HDS was calculated from the reported consumption of eight different food groups, designated as either favorable (fish, fruit, vegetables, and whole grains) or unfavorable (red or processed meats, sweets, sweetened beverages, and fried potatoes) (**Online Resource 2**). The consumption of each food group was categorized into quartiles, with ascending values (0–3) for favorable food groups and descending values (3–0) for unfavorable food groups. The sum of these values generated the HDS (with a range from 0 to 24, for which higher scores represent a healthier diet).

### Statistical analysis

Of the 4,344 participants who were eligible for the current study, we excluded 401 with incomplete diet data (missing data on portion sizes and/or missing data on >10% of the FFQ), six with an energy intake above 5,000 kcal per day, and 22 with incomplete tobacco data. Three hundred-thirty participants with a food intake level (FIL) below the 5th or above the 97.5th percentile were also excluded to alleviate the possible effects of dietary misreporting, especially underreporting. (Nota bene: FIL is the ratio between the reported energy intake and the estimated basal metabolic rate, for which values below 1.20 to 1.35 indicate underreporting [29].) Finally, to separate the dietary association of snus use from that of tobacco smoking, since our aim was to differentiate the two tobacco habits from each other, the 188 participants who reported current use of both snus and smoking tobacco were excluded in the main analysis (**Online Resource 3**). In a sensitivity analysis, the participants with cocurrent use of snus and smoking tobacco were reincluded to examine if their dietary habits differed from those with exclusive use of either tobacco product.

To limit the influence of outliers and to avoid the traditional assumptions of a linear regression model, we used quantile regression models to calculate the median difference in the HDS, intake of different food groups (in servings/week), and intake of macronutrients (as estimated percentages of energy intake) by tobacco use (never [reference], past, current snus, and current smoking). In subgroup analyses, snus users were further categorized by smoking history (never or past) and intensity of snus use (< or ≥4 cans/week [one can contains up to 42 grams of tobacco]).

Potential confounders were decided upon a priori based on their possible associations with tobacco use as well as with dietary habits, and the multivariable models included age (≤35, 35–44, 45–54, 55–64, and ≥65 years), examination year (1994, 1999, 2004, 2009, and 2014), educational level (university and non-university), BMI (<25, 25–29, and ≥30 kg/m^2^), diabetes (no and yes), and energy intake (quartiles of kcal). Further adjustment for civil status (married or cohabiting, other) had negligible influence on the results (data not shown). Missing data on covariates were handled using complete case analysis.

Statistical significance was set at a 2-sided *P*-value less than 0.05. All analyses were conducted using SPSS, version 27 (IBM Corp., Armonk, NY, USA).

## Results

A total of 3,397 men were included in the study (median age 51.1 years; 56.5% examined in 2004 to 2014) (**Online Resource 3**), of whom 21.0% were current snus users, 14.3% were current smokers, and 27.2% were past tobacco users. The prevalence of current snus use increased over time, from 17.6% in 1994 to 25.7% in 2014 (**Online Resource 4**). Around half of the current snus users had smoked in the past, and the majority of them consumed less than 4 cans per week (Table 1). Compared with never tobacco users, current snus users were slightly younger, had a lower energy intake from the diet, had less often a university education, and had more often a BMI of at least 30 kg/m^2^. Current snus users also had the lowest FIL-values, indicating the highest degree of dietary underreporting.

**Table 1:** Characteristics of the study population according to tobacco use (*n* = 3,397)

Characteristics	Tobacco use			
	Never	Past	Current, snus^a^	Current, smoking^b^
Participants (*n*)	1,271	927	713	486
Tobacco use (%)^c^				
Past use of snus	—	51.3	—	23.2
Past use of smoking tobacco	—	80.4	55.5	—
Covariates^d,e^				
Age (median, years)	48.1 (25.7)	57.4 (19.0)	45.8 (21.9)	53.2 (21.6)
Examined in 2004 to 2014 (%)	59.3 (56.6, 62.0)	57.7 (54.5, 60.9)	58.3 (54.7, 61.9)	44.0 (39.7, 48.5)
University education (%)	26.6 (24.2, 29.0)	20.7 (18.2, 23.4)	16.5 (13.9, 19.3)	16.8 (13.7, 20.3)
Energy intake (median, kcal/day)	1,912 (738)	1,861 (714)	1,809 (736)	1,857 (806)
Food intake level (median)^f^	1.06 (0.44)	1.03 (0.43)	0.97 (0.42)	1.04 (0.48)
Body mass index ≥30 kg/m^2^ (%)	15.7 (13.7, 17.7)	18.7 (16.3, 21.3)	18.7 (15.9, 21.6)	17.3 (14.1, 20.8)
Diagnosis of diabetes (%)	3.3 (2.4, 4.4)	6.8 (5.3, 8.6)	3.1 (2.0, 4.6)	4.1 (2.6, 6.2)

a 70.9% consumed less than 4 cans per week, and 29.1% consumed 4 or more cans per week.

b Includes current and occasional users as well as the following types of smoking tobacco: cigarettes, pipe, cigars, and cigarillos.

c Percentages might sum up to more than 100%, since some participants had used both snus and smoking tobacco.

d Based on complete data: 18 participants had missing data on educational level and 13 participants on diabetes status.

e Numbers in parentheses represent interquartile spread (continuous variables) or 95% confidence intervals (categorical variables).

f The ratio between the reported energy intake and the estimated basal metabolic rate.

The median values of the HDS, weekly servings of food groups, and estimated percentages of energy intake from different macronutrients according to tobacco use are shown in Table 2; and the quantile regression-based results are shown in **Online Resource 5** (unadjusted estimates) and Table 3 (adjusted estimates). In the multivariable-adjusted model (Table 3), compared with never tobacco users, current snus users had a lower HDS (*P* <0.001) and a lower consumption of fruits and berries (*P* = 0.001). In addition, their estimated energy intake consisted of less carbohydrates (*P* < 0.001) and of more total fat, saturated fat, monounsaturated fat, trans fat, and alcohol (*P* = 0.005, <0.001, <0.001, 0.013, and 0.034, respectively). Current smokers also had an unhealthier diet than never tobacco users, slightly more so than current snus users (Table 3). (Nota bene 1: in a direct comparison between the two tobacco habits, current smoking was associated with lower vegetable consumption [*P* = 0.022] and higher alcohol intake [*P* = 0.012].) (Nota bene 2: the dietary habits of cocurrent users of snus and smoking tobacco are shown in **Online Resource 6**, among whom the magnitude of the aforementioned associations seemed even larger.)

**Table 2:** Median values (interquartile spread) of dietary variables according to tobacco use

Dietary variables	Tobacco use			
	Never	Past	Current, snus	Current, smoking
Healthy diet score^a^	12 (6)	13 (6)	11 (5)	11 (6)
Food groups (servings/week)^b^				
Whole grains	15.14 (15.00)	17.24 (15.40)	13.04 (13.52)	14.58 (14.23)
Fruits and berries	6.58 (8.44)	7.60 (8.96)	5.62 (7.52)	5.62 (7.92)
Vegetables	7.58 (7.42)	7.56 (8.14)	7.02 (7.80)	6.52 (7.82)
Fish	1.14 (0.96)	1.14 (1.08)	1.12 (0.98)	1.14 (0.96)
Red or processed meat	7.88 (5.28)	7.88 (5.80)	7.86 (5.13)	7.99 (5.14)
Fried potatoes	1.00 (0.54)	0.58 (0.56)	1.00 (0.96)	0.98 (0.54)
Sweets	7.56 (9.78)	7.14 (12.48)	7.02 (12.89)	8.69 (16.22)
Sweetened drinks	2.94 (5.30)	1.98 (4.46)	2.56 (5.30)	2.56 (5.86)
Macronutrients (% of daily energy intake)^c^				
Carbohydrates	47.35 (9.25)	47.65 (10.04)	45.57 (9.89)	46.89 (9.09)
Protein	14.39 (2.98)	14.32 (2.65)	14.35 (2.90)	14.16 (2.98)
Fat	36.23 (8.78)	35.62 (9.76)	37.54 (9.02)	36.36 (8.63)
Saturated fat	15.20 (4.78)	14.82 (4.97)	16.00 (4.88)	15.60 (4.16)
Monounsaturated fat	12.67 (3.97)	11.56 (3.96)	13.02 (3.87)	11.94 (3.89)
Polyunsaturated fat	5.22 (2.20)	4.98 (2.26)	5.35 (2.31)	4.80 (1.92)
Trans fat	0.69 (0.58)	0.68 (0.57)	0.73 (0.60)	0.83 (0.72)
Sugar	6.65 (4.69)	6.37 (4.96)	6.30 (5.03)	7.00 (5.81)
Alcohol	1.27 (2.47)	1.66 (2.62)	1.60 (2.31)	1.84 (2.61)

a Calculated from the reported consumption of eight different food groups, designated as either favorable (fish, fruit, vegetables, and whole grains) or unfavorable (red or processed meats, sweets, sweetened beverages, and fried potatoes). The consumption of each food group was categorized into quartiles, with ascending values (0–3) for favorable food groups and descending values (3–0) for unfavorable food groups. The sum of these values generated the healthy diet score (with a range from 0 to 24, for which higher scores represent a healthier diet).

b See **Online Resource 2** for the included food items of each food group.

c Calculated by multiplying the daily intake of each macronutrient (in grams) with its caloric value (e.g. 9 kcal per gram of fat) and dividing that number by the daily energy intake (in kcal).

**Table 3:** Multivariable-adjusted median differences (95% confidence intervals) of dietary variables according to tobacco use^a^

Dietary variables	Tobacco use			
	Never	Past	Current, snus	Current, smoking
Healthy diet score	Ref	0.18 (−0.25, 0.61)	**−0.86 (−1.32, −0.40)**	**−1.06 (−1.59, −0.53)**
Food groups (servings/week)				
Whole grains	Ref	0.15 (−0.82, 1.13)	−0.24 (−1.29, 0.80)	−1.07 (−2.28, 0.12)
Fruits and berries	Ref	−0.02 (−0.60, 0.56)	**−1.03 (−1.65, −0.40)**	**−1.64 (−2.35, −0.93)**
Vegetables	Ref	−0.05 (−0.59, 0.50)	−0.03 (−0.61, 0.56)	**−0.89 (−1.56, −0.23)**
Fish	Ref	0.03 (−0.04, 0.10)	−0.06 (−0.14, 0.02)	0.01 (−0.08, 0.10)
Red or processed meat	Ref	−0.11 (−0.49, 0.26)	0.28 (−0.12, 0.68)	−0.01 (−0.47, 0.45)
Fried potatoes	Ref	−0.06 (−0.12, 0.00)	0.02 (−0.05, 0.08)	0.05 (−0.03, 0.12)
Sweets	Ref	−0.09 (−0.78, 0.59)	0.10 (−0.64, 0.83)	0.82 (−0.02, 1.66)
Sweetened drinks	Ref	**−0.35 (−0.65, −0.05)**	−0.12 (−0.44, 0.20)	0.25 (−0.12, 0.62)
Macronutrients (% of daily energy intake)				
Carbohydrates	Ref	**−1.18 (−1.83, −0.54)**	**−1.43 (−2.12, −0.74)**	**−1.65 (−2.45, −0.86)**
Protein	Ref	0.08 (−0.14, 0.29)	0.04 (−0.18, 0.27)	0.14 (−0.12, 0.39)
Fat	Ref	0.45 (−0.19, 1.09)	**0.99 (0.30, 1.67)**	**1.05 (0.27, 1.83)**
Saturated fat	Ref	0.10 (−0.26, 0.45)	**0.67 (0.29, 1.05)**	**0.78 (0.34, 1.21)**
Monounsaturated fat	Ref	0.12 (−0.11, 0.34)	**0.44 (0.20, 0.68)**	**0.41 (0.13, 0.68)**
Polyunsaturated fat	Ref	**0.16 (0.01, 0.30)**	0.07 (−0.09, 0.22)	−0.02 (−0.20, 0.16)
Trans fat	Ref	0.01 (−0.01, 0.03)	**0.03 (0.01, 0.06)**	0.01 (−0.02, 0.03)
Sugar	Ref	−0.30 (−0.66, 0.06)	−0.08 (−0.47, 0.30)	0.05 (−0.39, 0.49)
Alcohol	Ref	**0.46 (0.28, 0.64)**	**0.21 (0.02, 0.40)**	**0.52 (0.30, 0.74)**

a Estimated from quantile regression models and adjusted for age (≤35, 35–44, 45–54, 55–64, and ≥65 years), examination year (1994, 1999, 2004, 2009, and 2014), educational level (university and non-university), body mass index (<25, 25–29, and ≥30 kg/m^2^), diabetes (no and yes), and energy intake (quartiles of kcal). Bold text indicates statistically significant differences (*P* < 0.05).

The direction of the association between snus use and dietary habits was similar in the subgroup analysis by history of smoking (Table 4). However, the magnitude was more pronounced in current snus users without a history of smoking (with the exception of energy intake from alcoholic beverages); among whom the consumption of whole grains and fish was also lower (*P* = 0.006 and 0.023, respectively). Likewise, in the subgroup analysis by intensity of snus use, the magnitude of the association was slightly more pronounced in current snus users who consumed 4 or more cans per week (Table 4).

**Table 4:** Multivariable-adjusted median differences (95% confidence intervals) of dietary variables for current snus users compared with never tobacco users and according to history of smoking and intensity of snus use^a^

Dietary variables	History of smoking		Intensity of snus use	
	No (*n* = 317)	Yes (*n* = 396)	<4 cans/week (*n* = 500)	≥4 cans/week (*n* = 213)
Healthy diet score	**−1.05 (−1.68, −0.43)**	−0.53 (−1.10, 0.05)	**−0.77 (−1.30, −0.24)**	**−0.91 (−1.66, −0.16)**
Food groups (servings/week)				
Whole grains	**−1.97 (−3.40, −0.55)**	0.45 (−0.84, 1.75)	0.21 (−0.97, 1.39)	**−2.00 (−3.67, −0.32)**
Fruits and berries	**−1.12 (−1.97, −0.28)**	**−0.92 (−1.69, −0.15)**	**−1.10 (−1.80, −0.41)**	−0.92 (−1.90, 0.07)
Vegetables	−0.11 (−0.89, 0.67)	0.08 (−0.63, 0.79)	0.01 (−0.64, 0.66)	−0.23 (−1.15, 0.69)
Fish	**−0.13 (−0.24, −0.02)**	0.02 (−0.08, 0.12)	−0.04 (−0.13, 0.05)	**−0.14 (−0.27, −0.01)**
Red or processed meat	0.28 (−0.26, 0.82)	0.30 (−0.19, 0.80)	0.20 (−0.25, 0.65)	0.57 (−0.06, 1.21)
Fried potatoes	0.04 (−0.05, 0.13)	−0.03 (−0.11, 0.06)	0.00 (−0.07, 0.08)	0.03 (−0.07, 0.14)
Sweets	−0.13 (−1.12, 0.87)	0.52 (−0.38, 1.43)	0.38 (−0.46, 1.21)	−0.26 (−1.44, 0.92)
Sweetened drinks	0.08 (−0.35, 0.51)	−0.30 (−0.69, 0.09)	0.00 (−0.36, 0.36)	−0.34 (−0.85, 0.17)
Macronutrients (% of daily energy intake)				
Carbohydrates	**−1.67 (−2.61, −0.73)**	**−1.22 (−2.07, −0.37)**	**−1.05 (−1.83, −0.28)**	**−1.96 (−3.06, −0.86)**
Protein	−0.09 (−0.40, 0.22)	0.11 (−0.18, 0.39)	0.06 (−0.19, 0.32)	0.02 (−0.34, 0.38)
Fat	**1.25 (0.35, 2.15)**	0.68 (−0.14, 1.50)	0.67 (−0.09, 1.43)	**1.66 (0.59, 2.73)**
Saturated fat	**0.86 (0.36, 1.37)**	0.45 (−0.01, 0.92)	**0.61 (0.18, 1.03)**	**0.77 (0.16, 1.37)**
Monounsaturated fat	**0.54 (0.22, 0.86)**	**0.31 (0.02, 0.61)**	**0.33 (0.07, 0.60)**	**0.64 (0.26, 1.02)**
Polyunsaturated fat	0.00 (−0.21, 0.21)	0.09 (−0.11, 0.28)	0.05 (−0.12, 0.23)	0.07 (−0.18, 0.31)
Trans fat	**0.06 (0.02, 0.09)**	0.02 (−0.01, 0.06)	**0.03 (0.00, 0.06)**	0.03 (−0.01, 0.07)
Sugar	−0.07 (−0.58, 0.45)	−0.12 (−0.59, 0.35)	−0.10 (−0.53, 0.32)	−0.05 (−0.66, 0.55)
Alcohol	0.12 (−0.13, 0.38)	**0.26 (0.03, 0.49)**	0.20 (−0.02, 0.42)	**0.35 (0.04, 0.66)**

a Estimated from quantile regression models and adjusted for the same variables as in Table 2. Bold text indicates statistically significant differences (*P* < 0.05).

## Discussion

In this cross-sectional study, which included 3,397 Swedish men from five population-based surveys, we observed that snus users had an unhealthier diet than never tobacco users, as exemplified by a lower HDS (a proxy for overall diet quality). Snus users also consumed less fruit and berries, and their estimated energy intake consisted of less carbohydrates and of more total fat, saturated fat, monounsaturated fat, trans fat, and alcohol.

An interesting finding from the secondary analyses—not to say surprising, given that cigarette smoking is associated with a poorer diet (both in our and previous studies [22, 23])—was that snus users who had no history of smoking seemed to have a slightly unhealthier diet than those who had previously smoked, including a lower consumption of whole grains and fish. A possible explanation to this finding is that snus is used by more than one-fifth of Swedish men to assist in smoking cessation (30, 31), and it could be that those who quit smoking also undertake other lifestyle changes and shift to a healthier diet. Another interesting finding from the secondary analyses was that the dietary habits also seemed slightly worse among snus users who consumed more than 4 cans per week. This could be a partial explanation to the finding in a previous Swedish study, in which the prevalence of obesity and the metabolic syndrome was associated with snus use only among high-dose consumers (32).

The effects of snus use on health outcomes are in general less clear than the effects of cigarette smoking (4). However, given the widespread use of snus in Sweden (around 20% of the adult male population in 2021) (1), even the smallest hazardous effects are of importance from a public health perspective. If using CVD as an example, several studies do suggest that snus use might increase the risk of CVD, more so for mortality than for incidence (as summarized in **Online Resource 1**), but the potential confounding effect of diet has never been accounted for. A poor diet is an established risk factor for CVD (17–19), with a plethora of studies available on the impact of indivdual food groups and macronutrients. For example, and of relevance to the findings in the current study, there are multiple reports on the inverse associations with consumption of fruit, whole grains, and fish (33–35) as well as on the positive associations with intake of saturated fat, trans fat, and alcohol (36, 37). Better adherence to healthy eating patterns—as defined by different diet quality indexes—has also been inversely associated with CVD in several studies (38, 39).

While our study confirms that diet fulfills the criteria to be considered a potential confounder in the association of snus use with CVD (as well as with other health outcomes related to diet), it should be noted that the magnitude of the observed dietary differences was modest in absolute terms. The differences in food groups ranged from 0.02 to 1.03 servings per week, the differences in macronutrients ranged from 0.03 to 1.43 percentage points of estimated energy intake, and the difference in the HDS was 0.86 points (1.05 points among those without a history of smoking). As an example, the alcohol intake was only slightly higher among current snus users (0.21 percentage points), but the newly published Nordic Nutrition recommendations do recommend everyone to avoid alcohol for public health purposes (40). It is, therefore, possible that the impact of the snus users’ dietary habits could be marginal to small—but still not negligible—with respect to different health outcomes.

Epidemiological data on the association between snus use and dietary habits are sparse but consistent with our findings. In a previous Swedish study (9,954 participants, including 839 exclusive snus users), it was reported that snus users were less likely to be daily consumers of fruit and berries than non-tobacco users (21). In that study, as well as in a previous Norwegian study (23,622 participants, including 4,918 occasional or daily snus users) (20), snus users also had less regular eating habits than non-tobacco users.

The main strength of our study was the relatively large number of participants, who were recruited from a region where snus use is a common tobacco habit, allowing us to conduct multiple subgroup analyses without losing too much statistical power. The Northern Sweden MONICA study has also had a high participation rate over time, limiting the potential impact of selection bias (41).

Some limitations must also be mentioned. First, because of the low percentage of female snus users in the Northern Sweden MONICA study (around 5%), we restricted our analyses to male snus users, thereby questioning the generalizability of our findings to women. However, given that previous studies on the association between cigarette smoking and dietary habits have shown highly similar results in men and women (22), it is possible that our findings can be applied to female snus users too. Second, we used self-reported tobacco data, which, inevitably, led to a certain degree of misclassification. However, the tobacco questions in the Northern Sweden MONICA study have been shown to be in good agreement with plasma concentrations of cotinine (the predominant nicotine metabolite) (42). Third, even though the FFQ in the Northern Sweden MONICA study has been shown to have a validity similar to that of other FFQs (25), the dietary data were also self-reported and, therefore, prone to misclassification. Indeed, based on the median FIL-values in our study (i.e. the ratio between the reported energy intake and the estimated basal metabolic rate), there was evidence of a general underestimation of the participants’ dietary habits, irrespective of tobacco use (FIL-values < 1.20 [29]). As a consequence, on the absolute scale, the observed median values of the HDS, food groups, and macronutrients should be interpreted with caution. However, the comparisons by tobacco use on the relative scale should be more robust, even though there were indications of a more pronounced underreporting of dietary habits among current snus users (median FIL-value 0.97) than among never tobacco users (median FIL-value 1.06), which, if anything, should have biased the dietary comparisons toward the null. Fourth, the FFQ in our study was unaltered between 1994 and 2014. While the homogenous FFQ made it possible to pool dietary data from different surveys, it hindered us to fully capture the foods and beverages available at the time of examination. Finally, as in all observational studies, we cannot rule out the possibility of unmeasured confounding.

In conclusion, we observed that snus users had an unhealthier diet than never tobacco users in this cross-sectional study of Swedish men. Future studies on the association of snus use with CVD incidence and mortality (as well as with other health outcomes) should, therefore, consider diet as a potential confounder.

## Data Availability

The data that support the findings of this study are available from Umeå University, Sweden, but restrictions apply to the availability of these data, which were used under license for the current study, and so are not publicly available. Data are, however, available from the authors upon reasonable request and with permission of the MONICA study steering committee at the Biobank Research Unit, Umeå University, Umeå, Sweden.
