# Supplementary material for: Dietary habits among snus users: a population-based cross-sectional study
Source: Food Nutr Res. 2023 Aug 21;67:10.29219/fnr.v67.9537. doi: 10.29219/fnr.v67.9537 (PMC10492228; doi:10.29219/fnr.v67.9537)
Supplement: Supplementary file 1 [file FNR-67-9537-s001.docx]

**Online Supplementary Material**

**Online Resource 1** Multivariable-adjusted risk ratios, hazard ratios, or odds ratios of CVD incidence and mortality by snus use (yes vs. no), based on published studies from Sweden (bold text denotes statistically significant findings [*p* < 0.05])

|  | | | First author (reference number)^a^ | | | | | | | | | |  |  | |  |
| --- | --- | --- | --- | --- | --- | --- | --- | --- | --- | --- | --- | --- | --- | --- | --- | --- |
|  | | |  |  |  |  |  |  |  |  |  |  |  | |  | |
| Endpoint | | | Hansson  (4) | Janzon  (5) | Haglund  (6) | Wennberg  (7) | Huhtasaari  (8) | Hergens  (9) | Arefalk  (10) | Hansson  (11) | Hansson  (12) | Hergens  (13) | Titova  (14) | | Byhamre  (15) | |
| MI | | |  |  |  |  |  |  |  |  |  |  |  | |  | |
|  | Incidence | | 0.86 | 1.05 | 0.77 | 0.82-1.25 | **0.58** | 1.0 | — | — | 1.04 | 1.02 | 0.95 | | — | |
|  | Mortality | | — | — | 1.15 | 1.12-1.24 | 1.50 | — | — | — | **1.28** | **1.32** | — | | — | |
| Stroke | | |  |  |  |  |  |  |  |  |  |  |  | |  | |
|  | Incidence | | 0.91 | 0.97 | 1.07 | — | — | — | — | 1.04 | — | — | 1.04 | | — | |
|  | Mortality | | — | — | 1.01 | — | — | — | — | **1.32-1.42** | — | — | — | | — | |
| Heart failure | | |  |  |  |  |  |  |  |  |  |  |  | |  | |
|  | Incidence | | — | — | — | — | — | — | **1.28-2.08** | — | — | — | 0.99 | | — | |
|  | Mortality | | — | — | — | — | — | — | — | — | — | — | — | | — | |
| Overall CVD | | |  |  |  |  |  |  |  |  |  |  |  | |  | |
|  | Incidence | | 0.91 | — | — | — | — | — | — | — | — | — | — | | — | |
|  | | Mortality | — | — | — | — | — | — | — | — | — | — | 1.03 | | **1.27** | |

CVD, cardiovascular disease; MI, myocardial infarction

^a^ See the reference list in the main manuscript for details of each reference

**Online Resource 2** Food groups included in the healthy diet score

|  | |  |
| --- | --- | --- |
| Food group | | Included food items |
| Favorable foods | |  |
|  | Fish | Lean fish (e.g., perch, bass, and cod), fatty fish (e.g., herring, whitefish, char, and salmon), and salted fish |
|  | Fruit | Apple, pear, peach, banana, orange and other citrus fruits, and berries (fresh or frozen) |
|  | Vegetables | White cabbage, root vegetables, carrot, tomato, cucumber, lettuce, spinach, broccoli, kale, and mixed frozen vegetables |
|  | Whole grains | Whole grain crisp bread, whole grain soft bread, porridge (i.e., oat flake, whole wheat, rye, or barley porridge), and fiber cereals (e.g., muesli and granola) |
| Unfavorable foods | |  |
|  | Red or processed meat | Minced meat, meat stew, steak, bacon, sausage as dish, hamburger, sausage on bread, meat on bread, liver pâté on bread, and offal (e.g., liver and kidney) |
|  | Sweets | Ice cream, chocolate, candy, sugar, honey, marmalade, jam, cookies, and pastry |
|  | Sweetened beverages | Soft drinks, soda, and juice |
|  | Fried potatoes | French fries and fried potatoes |

**Online Resource 3** Flowchart of the study population (abbreviations: FIL, food intake level; MONICA, Monitoring of Trends and Determinants in Cardiovascular Disease)

*
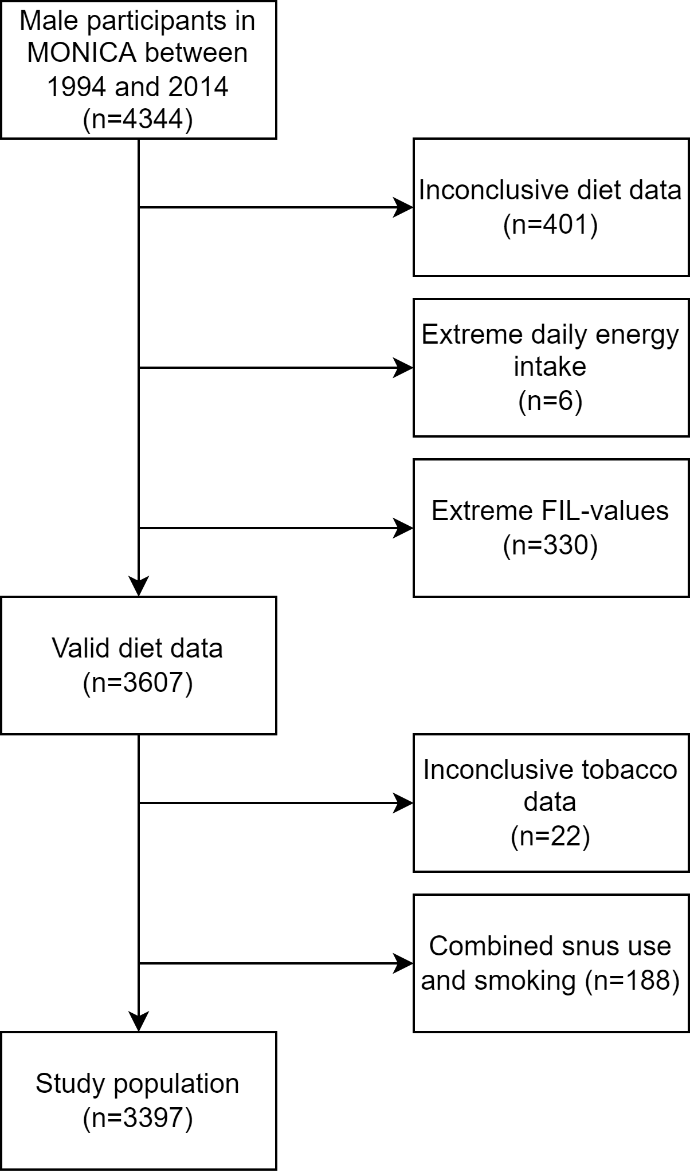
*

**Online Resource 4** Tobacco use according to examination year


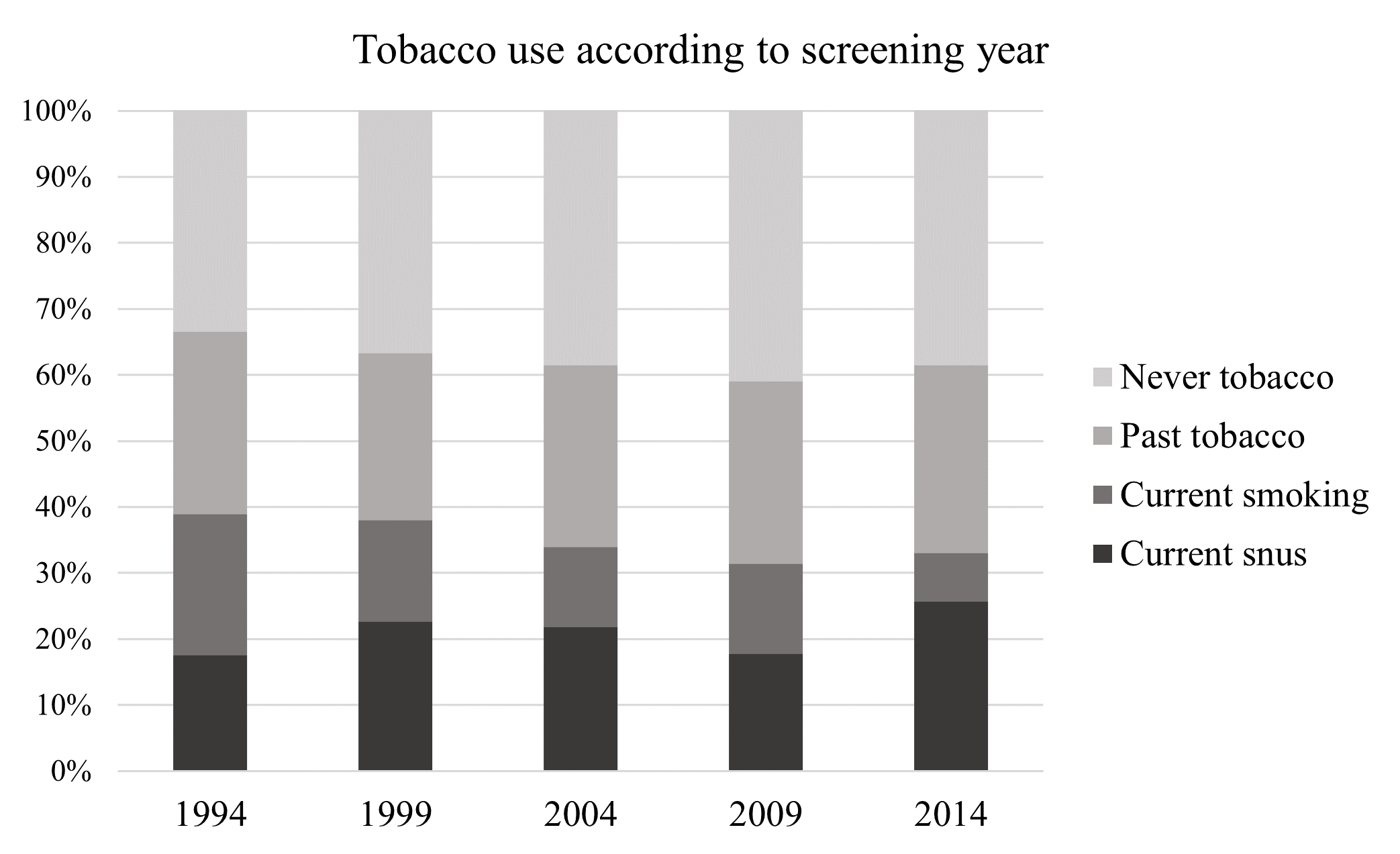


**Online Resource 5** Unadjusted median values of dietary variables according to tobacco use

|  | | Tobacco use | | | |
| --- | --- | --- | --- | --- | --- |
|  | |  |  |  |  |
| Dietary variables | | Never | Past | Current, snus | Current, smoking |
| Healthy diet score | | 12 | 13 | 11 | 11 |
| Food groups (servings/week) | |  |  |  |  |
|  | Whole grains | 15.14 | 17.24 | 13.04 | 14.58 |
|  | Fruits and berries | 6.58 | 7.60 | 5.62 | 5.62 |
|  | Vegetables | 7.58 | 7.56 | 7.02 | 6.52 |
|  | Fish | 1.14 | 1.14 | 1.12 | 1.14 |
|  | Red or processed meat | 7.88 | 7.88 | 7.86 | 7.99 |
|  | Fried potatoes | 1.00 | 0.58 | 1.00 | 0.98 |
|  | Sweets | 7.56 | 7.14 | 7.02 | 8.69 |
|  | Sweetened drinks | 2.94 | 1.98 | 2.56 | 2.56 |
| Macronutrients (% of daily energy intake) | |  |  |  |  |
|  | Carbohydrates | 47.35 | 47.65 | 45.57 | 46.89 |
|  | Protein | 14.39 | 14.32 | 14.35 | 14.16 |
|  | Fat | 36.23 | 35.62 | 37.54 | 36.36 |
|  | Saturated fat | 15.20 | 14.82 | 16.00 | 15.60 |
|  | Monounsaturated fat | 12.67 | 11.56 | 13.02 | 11.94 |
|  | Polyunsaturated fat | 5.22 | 4.98 | 5.35 | 4.80 |
|  | Trans fat | 0.69 | 0.68 | 0.73 | 0.83 |
|  | Sugar | 6.65 | 6.37 | 6.30 | 7.00 |
|  | Alcohol | 1.27 | 1.66 | 1.60 | 1.84 |
